# Efficacy and safety of Shenfu injection for patients with return of spontaneous circulation after sudden cardiac arrest: Protocol for a systematic review and meta-analysis

**DOI:** 10.1097/MD.0000000000012500

**Published:** 2018-09-21

**Authors:** Jiarong Ye, Zehao Zhu, Qianrong Liang, Xia Yan, Xiaotu Xi, Zhongde Zhang

**Affiliations:** a Guangdong Provincial Hospital of Chinese Medicine; b Guangzhou University of Chinese Medicine, The 2nd Clinical College; c Guangdong University of Foreign Studies, Guangzhou, China.

**Keywords:** protocol, return of spontaneous circulation, Shenfu injection, sudden cardiac arrest, systematic review

## Abstract

**Background:**

Sudden cardiac arrest (SCA) is one of the most common critical illnesses encountered in clinical practice. Shenfu injection (SFI) has received extensive attention as an alternative therapy that can effectively maintain the autonomic circulation function after cardiopulmonary resuscitation. However, the mechanism of SFI is not yet fully understood. In addition, there has been no systematic review or meta-analysis of SFI in the treatment of patients with return of spontaneous circulation after SCA. Herein, we describe the protocol of a proposed study based on the Preferred Reporting Items for Systematic Reviews and Meta-Analyses guidelines that aims to systematically evaluate the efficacy and safety of SFI in patients with return of spontaneous circulation after SCA.

**Methods:**

Two researchers will search 9 electronic databases (PubMed, Medline, Embase, Cochrane Library, Web of Science, China National Knowledge Infrastructure, Chinese VIP Information, Wanfang, and Chinese Biomedical Database) to identify all studies that meet the inclusion criteria and were published before July 2018. After information extraction and methodological quality evaluation, we will use Stata 13.0 software (STATA Corporation, College Station, TX, USA) to synthesize the data. The primary outcomes will be the survival rate and Glasgow Coma Scale.

**Results:**

The data synthesis results will objectively illustrate the efficacy and safety of SFI in patients with return of spontaneous circulation after SCA.

**Conclusion:**

The findings will provide a reference for the use of SFI in the treatment of patients with return of spontaneous circulation after SCA.

**Registration:**

PROSPERO (registration number: CRD42018104230).

## 1 Introduction

Sudden cardiac arrest (SCA) and sudden cardiac death (SCD) refer to the sudden termination of the mechanical activity of the heart with hemodynamic failure; these conditions are usually caused by persistent ventricular tachycardia or ventricular fibrillation (VF). If the autonomic cycle resumes as a result of an intervention (e.g., defibrillation) or automatic heart rate conversion, the event is called SCA (or SCD attempt); if the patient dies, the event is called SCD. The exact incidence of SCA in the United States is currently unclear, but is estimated to range from 180,000 to 450,000 annually.^[1,2]^ In North America and Europe, the estimated incidence of SCA in the general population is 50 to 100/100,000.^[3]^ The survival rate of SCA remains low, despite the development of Cardio Pulmonary Resuscitation (CPR), defibrillation, and other advanced resuscitation techniques over the past 50 years.^[4–8]^ Therefore, increasing the survival rate of such patients has become a popular research topic. In particular, there is a need for research into alternative and complementary treatments, especially in the field of traditional Chinese medicine.

Shenfu injection (SFI) is a patented traditional Chinese medicine that consists mainly of ginseng and aconite extracts. The active ingredient of ginseng is ginsenoside, while the main component of aconite is higenamine. Modern pharmacological studies have shown that ginsenoside has potential positive effects on heart disease through various properties, including anti-oxidation, reduction of platelet adhesion, regulation of vasomotor activity, improvement of lipid mass spectrometry, effects on various ion channels, and inhibition of the production and release of pro-inflammatory cytokines.^[9,10]^ In addition, higenamine strengthens myocardial contractility, relaxes the aorta, inhibits platelet aggregation, and has antithrombotic properties.^[11–13]^

Recent meta-analyses have shown that SFI combined with conventional therapy is more effective than conventional therapy alone in improving the heart function of patients with heart failure, regulating the blood pressure of patients with septic shock, and ultimately reducing mortality.^[14,15]^ Furthermore, SFI can significantly reduce cerebral ischemia-reperfusion injury after resuscitation by regulating neuronal mitochondrial function.^[16–18]^ Animal experiments have confirmed that SFI improves microvascular blood flow and coronary perfusion pressure during VF and CPR, reduces the required number of shocks and duration of CPR, and reduces energy exhaustion during VF.^[19,20]^ However, no meta-analysis has investigated the efficacy and safety of SFI in the treatment of SCA. Therefore, the proposed study was designed to evaluate the efficacy and safety of SFI combined with conventional therapy in patients with spontaneous circulation recovery after SCA, and thus provides a reference for the use of SFI in clinical practice.

## 2 Methods

### 2.1 Inclusion criteria for study selection

#### 2.1.1 Types of studies

The proposed study will include all randomized controlled trials evaluating the use of SFI in the treatment of patients with return of spontaneous circulation after SCA.

#### 2.1.2 Types of participants

All participants must have been diagnosed with SCA and recovered spontaneously after CPR. There will be no restrictions on the primary disease, disease duration, age, sex, or ethnicity.

#### 2.1.3 Types of interventions

The control group must have been treated using modern guidelines for routine treatment after CPR.^[21]^ The experimental group must have received SFI in addition to the treatment received by the control group.

#### 2.1.4 Outcomes

##### 2.1.4.1 Primary outcomes

Primary outcomes will be the survival rate and Glasgow Coma Scale.

##### 2.1.4.2 Secondary outcomes

Secondary outcomes will be the mechanical ventilation time, mean arterial pressure, blood oxygen saturation, and adverse events.

### 2.2 Search strategy

Nine databases will be searched to identify relevant randomized, controlled trials published before July 2018. Retrieval strategies will not be restricted by publication type, region, or language. The 9 databases will be: PubMed, Medline, Embase, Cochrane Library, Web of Science, China National Knowledge Infrastructure, Chinese VIP Information, Wanfang Database, and Chinese Biomedical Database. The search strategy used in the PubMed database will be formulated as follows:

#1 “cardiac arrest”[MeSH Terms] OR “cardiac arrest”[Title/Abstract] OR “sudden cardiac arrest”[Title/Abstract] OR “sudden cardiac”[Title/Abstract] OR “heart arrest”[Title/Abstract] OR “heart sudden arrest”[Title/Abstract] OR “cardio arrest”[Title/Abstract] OR “heart sudden pause”[Title/Abstract] OR “heart stop sudden”[Title/Abstract]
#2 “cardiopulmonary resuscitation”[MeSH Terms] OR “cardiopulmonary resuscitation” [Title/Abstract] OR “cardio pulmonary resuscitation”[MeSH Terms] OR “cardio pulmonary resuscitation”[Title/Abstract] OR “cardio pulmo resuscitation”[Title/Abstract] OR “the cardiopulmonary resuscitation”[Title/Abstract] OR “cardio-pulmonary resuscitation” [Title/Abstract] OR “cpr”[Title/Abstract] OR “CPR”[Title/Abstract]
#3 “return of spontaneous circulation”[MeSH Terms] OR “return of spontaneous circulation” [Title/Abstract] OR “spontaneous circulation restoration”[MeSH Terms] OR “spontaneous circulation restoration”[Title/Abstract] OR “restoration of spontaneous circulation”[Title/Abstract] OR “spontaneous circulation recovery”[Title/Abstract]
#4 “Shenfu injection”[MeSH Terms] OR “Shenfu injection”[Title/Abstract] OR “Shen-Fu injection”[Title/Abstract] OR “Sf injection”[Title/Abstract] OR “ginseng-monkshood extract”[Title/Abstract]
#5 (#1 OR #2 OR #3) AND #4

#### 2.2.1 Searching other resources

The reference lists of the retrieved literature will be further searched to identify any relevant grey literature. In addition, our members will read relevant medical journals and magazines to identify literature not included in the searched electronic databases.

### 2.3 Data collection and analysis

#### 2.3.1 Study selection

Two researchers will work together to complete the literature search. The same 2 researchers will screen the retrieved literature by reading the titles and abstracts. The full text of relevant literature will then be read, and the studies will be selected in accordance with the inclusion criteria.

#### 2.3.2 Data extraction

Two researchers will extract the necessary information based on the “Information Extraction Form” that has been prepared in advance, including the basic condition of the patient, treatment plan, observation indicators, and adverse reactions. In cases of disagreement, the 2 researchers will crosscheck and discuss the discrepancy, or seek advice from a third party.

#### 2.3.3 Risk of bias assessment

To assess the methodological quality of the included studies, our team will use the Cochrane risk of bias tool to examine 6 aspects: randomization of sequence generation, concealment of allocation methods, integrity of outcome data, selectivity of outcome reporting, and other sources of bias.^[22]^ We will try to obtain any missing information from the original authors when possible. Ultimately, the studies will be classified as having a low, unclear, or high risk of bias in accordance with the quality classification criteria. The methodological quality of each study will be independently assessed by 2 authors, and any disputes will be resolved through consultation with a senior author.

#### 2.3.4 Measures of treatment effect

Continuous variables will be weighted by the mean differences, while dichotomous variables will be compared using the odds ratios. The confidence intervals (CI) for all reported results will be set at 95%.

#### 2.3.5 Dealing with missing data

If data are missing from the literature, we will attempt to contact the corresponding author by phone or e-mail. If the contact fails, we will perform a limited analysis based on the existing data.

#### 2.3.6 Assessment of heterogeneity

Heterogeneity testing is required for the synthesis analysis of the same outcome assessed in multiple studies. The heterogeneity is assessed using the *I*^2^ or *P* values. Values of *I*^2^ ≥50% or *P* < .1 indicate the presence of significant heterogeneity, and so a random effect model will be selected. Conversely, the fixed effect model will be selected when there is a small degree of heterogeneity.

#### 2.3.7 Assessment of reporting bias

The presence of publication bias can be analyzed by funnel plots. If the points on both sides of the funnel plot are scattered and asymmetrical, there is a publication bias, and the reliability is low. In contrast, if there is basically symmetrical distribution of the points on both sides of the funnel plot, there is no publication bias, and the result is reliable.

#### 2.3.8 Data synthesis

The appropriate treatment effect will be chosen based on different variables. In accordance with the heterogeneity test results, the appropriate model (random or fixed) will then be selected to merge the outcome indicators. Differences are considered statistically significant if the *Z* test results indicate that *P* is <.05, and the 95% CI do not contain 1 (for dichotomous variables) or the 95% CI do not contain 0 (for continuous variables). When the difference is not statistically significant, sensitivity analysis and subgroup analysis will be performed to identify the source(s) of heterogeneity.

#### 2.3.9 Subgroup analysis

When heterogeneity is detected, the source of the heterogeneity will be determined by performing further subgroup analyses based on patient sex, age, disease type, drug dose, and course of treatment.

#### 2.3.10 Sensitivity analysis

If the primary outcome analyses involve a large number of included studies and a large degree of heterogeneity, the included studies will be investigated individually to increase the stability of the final results.

#### 2.3.11 Ethics and dissemination

All data included in this study are derived from published literature and do not include patient personal data, so no ethical approval is required. The final meta-analysis results will be published in a peer-reviewed journal.

## 3 Discussion

SCA is relatively common in clinical practice, and can occur due to a variety of disease processes. Furthermore, SCA accounts for the highest mortality rate in emergency patients.^[23]^ At present, CPR is the main treatment of SCA. However, the success rate of CPR is low, and the patient often cannot effectively maintain the autonomic cycle.^[24]^ Therefore, it is imperative to seek more effective and safe treatments for SCA.

The recent wide application of traditional Chinese medicine has revealed that many traditional Chinese medicines can strengthen the body and promote recovery. Among them, SFI plays a huge role in the treatment of critically ill patients and those with severe diseases. Clinical studies have shown that SFI combined with conventional treatment can effectively maintain the spontaneous circulation of patients after successful CPR, with minimal adverse effects.^[25]^ The mechanism by which SFI improves the cardiovascular circulation is not completely clear. Therefore, we plan to conduct a systematic review and meta-analysis of existing clinical studies to objectively evaluate the efficacy and safety of SFI in the treatment of patients with self-circulatory recovery after SCA. However, the proposed study has certain limitations; there is no guarantee that consistent criteria will have been used to assess all outcomes in included studies, and individual differences in populations of different ethnicities may affect the final study results.

## Acknowledgments

The authors thank Dr. Kelly Zammit, BVSc, from Liwen Bianji, Edanz Group China (www.liwenbianji.cn/ac), for editing the English text of a draft of this manuscript.

## Author contributions

This manuscript is completed by Jiarong Ye and Zehao Zhu. Study retrieval and data curation are handled by Jiarong Ye and Qianrong Liang. In terms of methodology, we mainly consult Xiaotu Xi and Xia Yan. Zehao Zhu is responsible for software operations. Research costs are fully funded by Zhongde Zhang.

**Data curation:** Jiarong Ye, Qianrong Liang

**Funding acquisition**: Zhongde Zhang

**Methodology:** Xiaotu Xi, Xia Yan

**Software**: Zehao Zhu

**Writing – original draft**: Jiarong Ye, Zehao Zhu

Jiarong Ye orcid: 0000-0002-7512-5639

Zehao Zhu orcid: 0000-0003-0293-8986

Qianrong Liang orcid: 0000-0003-0801-4557

Xia Yan orcid: 0000-0001-6144-9336

Xiaotu Xi orcid: 0000-0002-0711-4137

Zhongde Zhang orcid: 0000-0002-7909-8877
